# Probing the origin of excitonic states in monolayer WSe_**2**_

**DOI:** 10.1038/srep22414

**Published:** 2016-03-04

**Authors:** Jiani Huang, Thang B. Hoang, Maiken H. Mikkelsen

**Affiliations:** 1 Department of Physics, Duke University, Durham, North Carolina, 27708, USA; 2 Department of Electrical and Computer Engineering, Duke University, Durham, North Carolina, 27708, USA

## Abstract

Two-dimensional transition metal dichalcogenides (TMDCs) have spurred excitement for potential applications in optoelectronic and valleytronic devices; however, the origin of the dynamics of excitons, trions, and other localized states in these low dimensional materials is not well-understood. Here, we experimentally probed the dynamics of excitonic states in monolayer WSe_2_ by investigating the temperature and polarization dependent photoluminescence (PL) spectra. Four pronounced PL peaks were identified below a temperature of 60 K at near-resonant excitation and assigned to exciton, trion and localized states from excitation power dependence measurements. We find that the localized states vanish above 65 K, while exciton and trion emission peaks remain up to room temperature. This can be explained by a multi-level model developed for conventional semiconductors and applied to monolayer TMDCs for the first time here. From this model, we estimated a lower bound of the exciton binding energy of 198 meV for monolayer WSe_2_ and explained the vanishing of the localized states. Additionally, we observed a rapid decrease in the degree of circular polarization of the PL at increasing temperatures indicating a relatively strong electron-phonon coupling and impurity-related scattering. Our results reveal further insight into the excitonic states in monolayer WSe_2_ which is critical for future practical applications.

Monolayer transition metal dichalcogenides (TMDCs), a new class of two-dimensional (2D) materials analogous to graphene, have received considerable attention in recent years due to their unique optical and electronic properties^1^,^2^,^3^. Unlike other semiconductors and few-layer TMDCs, electrons and holes in monolayer TMDCs are tightly bound together at the energy degenerate ±K valleys, as a consequence of reduced dielectric screening effect and strong Coulomb interactions^4^,^5^, giving rise to valley excitons and trions. The exciton binding energy of monolayer TMDCs has been predicted to be in the range of 0.5 eV to 1 eV^4^,^6^, which is more than one order of magnitude larger than conventional semiconductors such as GaAs^7^,^8^. Due to this large binding energy, excitons remain stable even at room temperature and hence is predicted to play a key role in future optoelectronics and valleytronics applications^3^. Determination of the binding energy is also critical to provide insight into other physical properties of excitons such as the Bohr radius and many-body interactions. To date, a number of experimental techniques have been used to determine the exciton binding energy, however, the obtained values from theoretical calculations^6^,^9^ and different experimental methods^5^,^10^,^11^,^12^ are inconsistent, and may also depend upon specific sample preparation conditions.

In addition to the determination of exciton binding energies of monolayer TMDCs, the control of valley exciton and trion dynamics is also of great importance and has been widely explored by various experimental methods, such as electrical gating^13^, optical pumping^14^,^15^ and the application of a magnetic field^16^. However, our understanding of the fundamental properties of the intricate excitonic features in monolayer TMDCs remains incomplete. Various possible mechanisms, including impurity-related scattering, interaction with phonons and carrier-carrier interactions, still need to be systematically addressed. Moreover, the optical studies of excitonic features that have been reported so far^17^,^18^,^19^,^20^,^21^, are not consistent with each other and also show striking material variations.

Here, we experimentally investigated the evolution of multiple PL emission peaks in monolayer WSe_2_ in order to provide further insight into the behavior and origin of the excitonic and localized states in monolayer TMDCs as well as their associated binding energies. A multi-level model commonly employed for conventional semiconductors such as zinc oxide nanowires^22^, was employed here to describe the temperature-dependent behavior of excitonic and localized states and reveal a lower bound on the exciton binding energy in monolayer WSe_2_. At a temperature of *T* = 10 K, we observed a clear neutral exciton emission at ~1.75 eV and trion emission at ~1.72 eV, yielding a large trion binding energy of ~30 meV. Temperature-dependent PL measurements showed that both the exciton and trion emissions existed at room temperature as a result of their large binding energies, whereas other localized, defect-related emission states vanished above *T* = 65 K. The evolution of the PL emission with temperature in monolayer WSe_2_ revealed a combined effect of large binding energies and strong electron-phonon interactions. Moreover, an observed difference in the temperature-dependent degree of circular polarization between WSe_2_ and MoS_2_ indicated stronger electron-phonon coupling and impurity-related scattering in monolayer WSe_2_.

For these studies, monolayer WSe_2_ flakes were exfoliated from its bulk crystal (2D Semiconductors Inc.) onto a 285 nm SiO_2_/Si substrate using the well-established micro-mechanical exfoliation technique^23^. The thickness of the SiO_2_ layer was chosen to offer the best optical contrast between thin WSe_2_ flakes and the substrate, thus increasing the visibility of the single-layer sheets^24^. After promising thin flakes were identified under an optical microscope, as shown in Fig. 1(a), and confirmed by Raman spectroscopy with a typical Raman mode at ~250 cm^−1^ for single-layer WSe_2_^25^, we further used atomic force microscopy (AFM) to measure the layer thickness. Figure 1(b) shows the AFM image of the area indicated in the optical image in Fig. 1(a). The height profile shown in Fig. 1(c) was taken along the white dashed line in Fig. 1(b), revealing a thickness of 0.7 nm for monolayer WSe_2_, in agreement with previous studies^25^,^26^.

For low temperature measurements, the sample was mounted in a cryostat (Janis ST-500 modified with an extension snout) cooled by liquid helium and all of the following measurements were carried out in a confocal microscopy set-up. The experiments were performed using two different excitation lasers. One was a 488 nm continuous wave (cw) Argon laser, and the other was a femtosecond laser at 632 nm generated by a tunable frequency-doubled optical parametric oscillator (OPO) pumped by a Ti^3+^:sapphire pulsed laser. For the experiments requiring circularly polarized excitation, the laser first passed through a Glan-Thompson polarizer and then a broadband quarter waveplate. The laser power was maintained below 30 μW, which is in the linear absorption regime as shown in Fig. 2, to avoid any heating or saturation effects. The laser beam was focused onto the sample via a 50×, 0.65 NA Nikon microscope objective with a laser spot size of ~1 μm. To identify the right-handed (*σ*^+^) and left-handed (*σ*^*−*^) circularly polarized PL signals, the emission from the sample was sent through a quarter waveplate followed by a linear polarizer. The beam was then focused at the entrance slit of a spectrometer and detected by a charge-coupled device (CCD) camera. Scattered laser light was blocked by a suitable long-pass filter placed immediately before the spectrometer entrance slit.

PL spectra from monolayer and bulk WSe_2_ flakes were first measured at a temperature of *T* = 10 K using the 488 nm cw excitation laser. Five pronounced PL peaks were observed from monolayer WSe_2_ at low temperature as displayed in the inset of Fig. 2(a), which was significantly different from the spectral characteristics of WSe_2_ monolayers at room temperature where only one broad peak was observed at 1.65 eV^25^,^26^. Additionally, a PL spectrum from bulk WSe_2_ taken at a temperature of *T* = 10 K is also shown in the inset. Compared with the PL spectrum from the monolayer, the red-shifted, weak PL indicates the transition to an indirect bandgap in bulk WSe_2_. The origin of the multiple emission peaks in monolayer WSe_2_ is further discussed below by observing the excitation power dependence and the evolution of the PL spectra from *T* = 10 K to room temperature. The excitation power dependence of the five PL peaks from monolayer WSe_2_ at *T* = 10 K is shown in Fig. 2, where the peaks are labeled as indicated in the inset. The solid lines are fits to the data using a power law: *I* ∝ *P^α^*, where *I* is the PL peak intensity for a given excitation power, *P*. The extracted exponent factor, *α,* for the five peaks was 1.0, 1.0, 1.6, 1.3 and 0.8, in the order from peak 1 to 5, revealing insight into different dominating recombination processes for each peak.

At *T* = 10 K, peaks 1 and 2 are primarily attributed to the radiative recombination of excitons and trions^16^,^17^,^19^,^27^, respectively, because the photon emission rate was observed to be linearly dependent on the excitation power (*I* ∝ *P*). This linear dependence is expected from the first-order rate equation for the radiative recombination process^28^. For peaks 3 and 4, several earlier reports have observed similar emission features, however, the nature of these peaks appear to depend on specific experimental conditions. While Wang *et al*.^17^ and Jones *et al*.^27^ assigned these peaks as localized states, You *et al*.^19^, under much higher pulsed laser excitation, have demonstrated the observation of biexciton emission. In our experiment, both peaks 3 and 4 were observable under cw excitation even with relatively low power (<30 μW) as shown in the low-temperature PL spectrum in the inset in Fig. 2(a), thus defect-related localized state transitions is the most likely origin of these peaks. When photo-excited electron-hole pairs are trapped in potential wells, which may be created by lattice defects or residual impurities commonly introduced during the mechanical exfoliation process^29^, localized states may form within the bandgap with an emission energy below the exciton and trion emission energies. An exponent factor *α* between 1 and 2 is expected from these bound exciton transitions^28^ which is consistent with our observations. Several mechanisms could explain the sub-linear power dependence of peak 5 17,18,27,30. Among those explanations, phonon side-band emission is one possible mechanism^27^, which can be interpreted as the radiative recombination of electrons and holes separately localized at different spatial sites^31^. Considering the rate equations for free and localized carriers, one can derive that the PL intensity arising from the recombination of localized electron-hole pairs is *I* ∝ *P*^0.5^ 31. Abundant defects and impurities common in exfoliated monolayer WSe_2_ may explain the slightly larger extracted exponent factor of 0.8 for peak 5 than predicted theoretically for phonon sideband emission. An alternative explanation for the origin of peak 5 may be excitons bound to isoelectronic defects in the silicon substrate^30^, where isoelectronic traps dominate the recombination process.

The polarization of the PL offers opportunities for optically manipulated valley index in monolayer TMDCs. However, the degree of PL polarization under 488 nm (~2.54 eV) excitation was relatively low at ~10% even at *T* = 10 K, because the excitation was far from the exciton emission energy of monolayer WSe_2_ as shown above^14^. Thus, in all of the following measurements, the excitation was performed using a 632 nm pulsed laser (~1.96 eV), which is closer to the neutral exciton emission energy, thus leading to a larger degree of PL polarization at low temperatures. Under this excitation, three pronounced peaks were identified at a temperature of *T* = 10 K and shown in Fig. 3(a) along with the evolution of the PL spectra with increasing temperature. In the temperature range between 30 and 50 K, four PL peaks were clearly observed. Peak 1 at ~1.75 eV and peak 2 at ~1.72 eV were recognized as the neutral exciton and trion emission, respectively, which were consistent with previous reports^27^ and the cw excitation measurement presented above. A trion binding energy of ~30 meV is obtained from a clear separation between the exciton and trion peaks. Because of this relatively large trion binding energy, trions could theoretically survive at room temperature where the thermal energy is *k*_*B*_*T* ~25 meV. The other two peaks with lower photon energies were recognized as localized states. As the temperature increased, all of the peaks red-shifted, and followed each other closely representing a decreased bandgap. The intensity of peaks 3 and 4 significantly decreased above a temperature of 50 K and eventually vanished, whereas clear exciton and trion peaks remained in the temperature range between 50 and 150 K. As the temperature further increased beyond 150 K, the neutral exciton peak dominated the PL spectrum with a long low-energy tail, which may be a signature of the existence of trion emission at room temperature. The main exciton emission peak at room temperature is because of an extremely large exciton binding energy of monolayer WSe_2_ compared with conventional semiconductors^5^; this means that electrons and holes are tightly bound together and can hardly escape due to thermal fluctuations. Conversely, other peaks have smaller binding energies, which can be thermalized more easily with increasing temperatures.

To further clarify the underlying dynamics responsible for the evolution of the emission peaks, we fitted the multiple PL peaks to an integrated Lorentz function and then extracted the photon energies of peaks 1, 2 and 4 as a function of temperature, as shown in Fig. 3(b). The evolution of peak 3 is not shown here because it only existed at three measured temperatures. The solid lines are fits to a modified Varshni’s equation describing the temperature dependence of a semiconductor bandgap^13^,^32^,^33^:


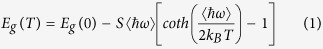


where *E*_*g*_(0) is the transition energy at *T* = 0 K, *S* is a dimensionless constant describing the strength of the electron-phonon coupling, and 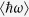 represents the average acoustic phonon energy involved in electron-phonon interactions. From the fits, we extracted *S* ≈ 2.33, 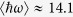 meV for the exciton, and *S* ≈ 2.32, 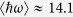 meV for the trion. This electron-phonon coupling constant *S* of monolayer WSe_2_ is larger than reported for other monolayer TMDCs, such as MoS_2_ (*S* ≈ 1.82) and MoSe_2_ (*S* ≈ 1.93)^32^. This difference may originate from the relatively smaller effective mass in the intervalley transition for monolayer WSe_2_^34^, thus leading to a stronger electron-phonon coupling. Additionally, it should be noted that the exciton-phonon interaction also plays a significant role in the shift of the exciton peak energies^35^. When the exciton moves within the crystal lattice, it interacts with phonons via scattering processes. At low temperatures, phonons are relatively inactive; therefore, the scattering is primarily dominated by phonon absorption. As the temperature increases, phonon emission and absorption contribute equally to the exciton scattering; thus, the exciton energy shifts as a result of this exciton-phonon interaction^35^. It has also been calculated that beyond the Debye temperature, the exciton energy has a linear temperature dependence, whereas it is independent of temperature in the low temperature limit, which is consistent with the sole contribution of phonon absorption^35^. However, this calculation only provides a general trend and the actual dependence is largely associated with the material properties, which can also be modified by other effects such as thermal expansion and impurities in the material.

Further analysis of the temperature dependent data reveals insight into the exciton and trion binding energies. By fitting the four emission peaks into an integrated Lorentz function, we extracted the PL intensities of peaks 1, 2 and 4 labeled in Fig. 3(a), and plotted them as a function of 1/*T* as shown in Fig. 3(c). As temperature increased, the intensities of peaks 1 and 2 first gradually increased then dramatically decreased, whereas the intensity of peak 4 only decreased with temperature. The multi-level model for the temperature dependence of the PL peak intensities is given by^22^,^36^,^37^:


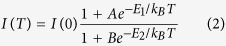


where *I*(0) is the PL intensity at *T* = 0 K, *k*_*B*_ is the Boltzmann constant, and *A* and *B* are fitting parameters. *E*_*1*_ describes the activation energy that causes the increase in PL intensity with increasing temperature, whereas *E*_*2*_ represents the activation energy for the normal thermal quenching process at higher temperatures. By fitting the experimental data, we obtained *E*_1_ ≈ 0.1 meV, *E*_2_ ≈ 198 meV for the exciton, and *E*_1_  ≈ 3 meV, *E*_2_ ≈ 54 meV for the trion. The value of *E*_*2*_ for the exciton represents the thermal energy that is needed for the normal thermal quenching process as the temperature is increased up to 300 K, which is smaller than the previously reported exciton binding energy of 370 meV for monolayer WSe_2_ obtained from two-photon PL excitation spectroscopy^5^ and 790 meV obtained from optical reflectivity/absorption spectra^12^. Since our sample has not been completely thermally quenched yet, the *E*_*2*_ value obtained here only represents a lower bound of the exciton binding energy of monolayer WSe_2_. An additional reason for the discrepancy could be variations in the number of impurities and defects in our exfoliated sample as well as interactions between the carriers and the heavily doped silicon substrate.

As noted above, peaks 3 and 4, corresponding to localized states, were easier to thermalize than the exciton and trion states. At low temperatures, a certain amount of carriers could be captured by these localized, trapped states. As temperature increased, the trapped carriers could be released again from the localized states and recombine radiatively, leading to the increased PL intensity in the exciton and trion peaks below *T* = 60 K. Moreover, between 50 K and 100 K, the trion peak even surpassed the exciton peak because carriers from peaks 3 and 4 were thermalized into the trion peak, resulting in a dramatic increase in its PL intensity. The value of *E*_*1*_ for the trion (~3 meV) was consistent with the temperature point of *T* ≈ 65 K where peaks 3 and 4 vanished in the measured PL spectrum.

Next, we turn our attention to the circular polarization of the emission peaks and their evolution with increasing temperatures. The degree of circular polarization of peaks 1, 2 and 4 as a function of temperature is shown in Fig. 4. Here, the degree of circular polarization is given by^14^,^15^:


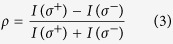


where *I*(σ^+^) and *I*(σ^−^) correspond to the PL intensity of the σ^+^ and σ^−^ polarization components, respectively. As observed in Fig. 4, under near-resonant circularly polarized excitation, the trion peak generally has a larger degree of circular polarization than the exciton peak; however, they both follow a similar trend with increasing temperature. While the exciton and trion emissions show a relatively large value of 25 % and 37 %, the localized states also show a small circular polarization of 13% at a temperature of *T* = 10 K. The observed circular polarization of these localized states is consistent with previous reports^17^,^27^, however, the origin is presently not well-understood. One possible mechanism could be related to a partial transfer of the valley polarization from optically generated electron-hole pairs to the localized electrons or holes^38^. Additionally, we also observed variations in the degree of circular polarization between monolayer WSe_2_ and MoS_2_. In contrast to monolayer MoS_2_, which displays a flat plateau with a circular polarization of ~31% below *T* = 90 K^15^, the degree of circular polarization of monolayer WSe_2_ dramatically decreased at temperatures below *T* = 50 K for both the exciton and trion peak. Beyond *T* = 50 K, the circular polarization gradually reduced, indicating the domination of phonons in the intervalley scattering at high temperatures. As revealed from the fitting parameters in the modified Varshni’s equation (1), the electron-phonon coupling strength *S* of monolayer WSe_2_ is stronger than that of monolayer MoS_2_ due to its relatively smaller effective mass in the intervalley transitions. As a consequence, the difference in the degree of circular polarization between WSe_2_ and MoS_2_ is likely due to the relatively stronger electron-phonon coupling and lower Debye temperature of monolayer WSe_2_, causing phonons to be involved in the intervalley scattering at much lower temperature than in monolayer MoS_2_. Therefore, the degree of circular polarization of monolayer WSe_2_ displayed a significant drop below *T* = 50 K as opposed to showing a similar temperature independence to monolayer MoS_2_. Moreover, abundant impurities and vacancies, as well as the effect of the heavily doped substrate, also play roles in determining the degree of circular polarization, which may account for the absence of a plateau at low temperatures. Finally, we applied a small in-plane magnetic field of ~0.35 T to the WSe_2_ monolayer at *T* = 10 K. The degree of circular polarization did not show any visible change, which agrees with previous reports^15^ and further demonstrates that we are indeed probing the valley polarization in monolayer WSe_2_.

To summarize, we have experimentally investigated the PL spectra from mechanically exfoliated monolayer WSe_2_ and probed the dependence of the intensity and energy of the exciton and trion emission, as well as the localized states, with temperature and excitation power. Contrary to other members of monolayer TMDCs such as MoS_2_ and MoSe_2_, the temperature dependence of the valley polarization in monolayer WSe_2_ under near-resonant circularly polarized excitation lacks a flat plateau at low temperatures, which indicates a stronger electron-phonon coupling and impurity-related scattering in monolayer WSe_2_. We have also successfully applied a multi-level model developed for conventional semiconductors to monolayer TMDCs, which explains the dynamics of various excitonic states and revealed a lower bound for the exciton binding energy. The insight into the excitonic and localized states in monolayer WSe_2_ provided by these experiments is an important step towards materials optimization for potential future optoelectronic device applications such as photodetectors and photovoltaic cells.

## Additional Information

**How to cite this article**: Huang, J. *et al*. Probing the origin of excitonic states in monolayer WSe_2_. *Sci. Rep.* **6**, 22414; doi: 10.1038/srep22414 (2016).

## Figures and Tables

**Figure 1:**
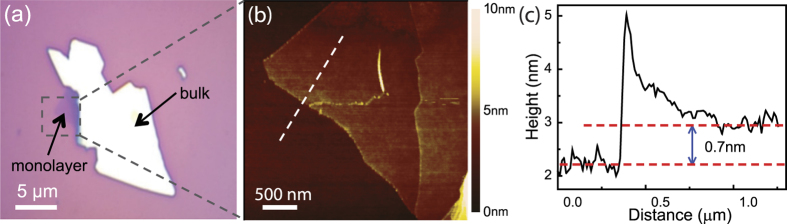
Optical and AFM images of a WSe_2_ sample. (**a**) Optical microscopy image taken with a 100× objective. (**b**) AFM image of the area in (**a**) indicated by the dashed lines. (**c**) Height profile taken along the dashed line in (**b**) confirming the presence of monolayer WSe_2_.

**Figure 2:**
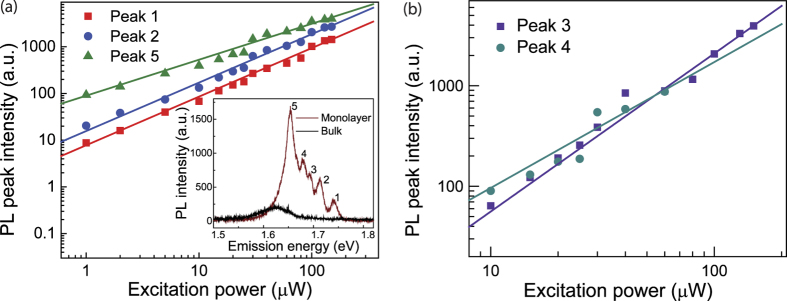
Excitation power dependence of emission dynamics. (**a**) PL intensity as a function of the excitation power for peaks 1, 2 and 5. Inset: PL spectra from monolayer and bulk WSe_2_ at *T* = 10 K. Five different emission peaks were observed for monolayer WSe_2_, whereas only one broad peak was observed for the bulk. A 30 μW, 488 nm cw excitation laser was used in the measurements. (**b**) PL intensity as a function of the excitation power for peaks 3 and 4. The solid lines are fits to a power law.

**Figure 3:**
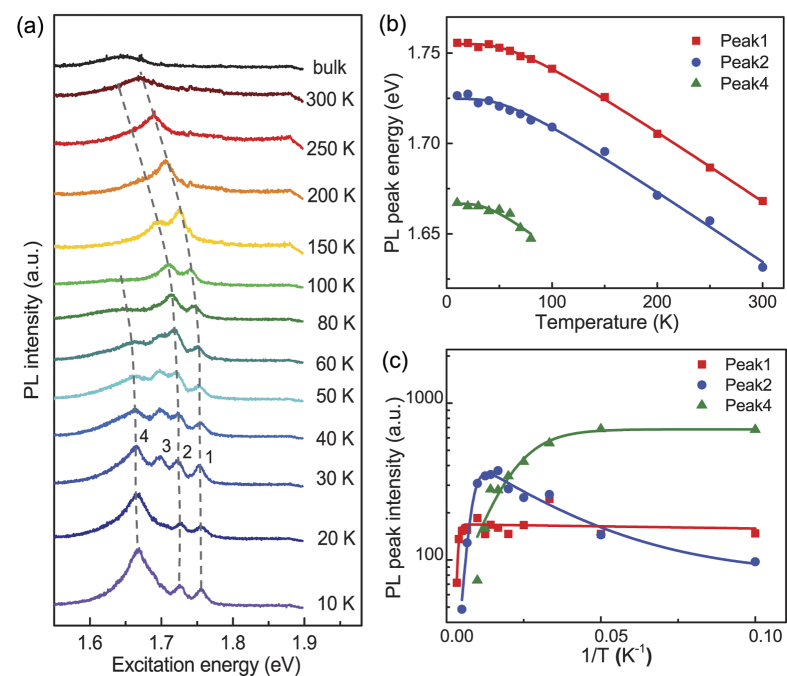
Temperature dependence of emission dynamics. (**a**) PL spectra of monolayer WSe_2_ at different temperatures. The top curve is bulk WSe_2_ at *T* = 300 K for comparison. The dashed gray lines are guides to the eye. (**b**) Photon energies of the peaks labeled as 1, 2, 4 in (**a**) as a function of temperature. The solid lines are fits to the data using Eq. (1). (**c**) PL intensities of peaks 1, 2 and 4 as a function of 1/*T*. The solid lines are fits to the data using Eq. (2). A 30 μW, 632 nm fs-laser was used for excitation in the measurements.

**Figure 4:**
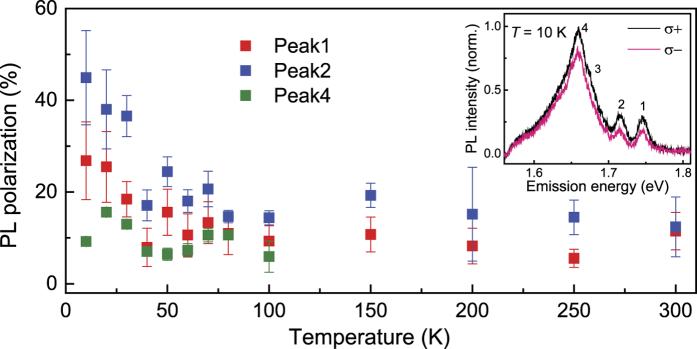
Temperature dependence of the PL polarization of monolayer WSe_2_. Degree of circular polarization as defined in Eq. (3) as a function of temperature for peaks 1, 2 and 4 as labeled in the inset. Inset: Polarization-resolved PL spectra for *σ*^+^ and *σ*^*−*^ detection for a 30 μW, 632 nm (1.96 eV) laser excitation at *T* = 10 K.
